# Addressing Psychological Distress in College Students Through Mindfulness Training: A Pre–Post Intervention Across Three Cohorts with Different Delivery Methods

**DOI:** 10.3390/ijerph22071027

**Published:** 2025-06-27

**Authors:** Rebecca Ciacchini, Silvia Villani, Mario Miniati, Graziella Orrù, Angelo Gemignani, Ciro Conversano

**Affiliations:** 1 Department of Surgical, Medical, Molecular Pathology and Critical Care Medicine, University of Pisa, 56126 Pisa, Italy; silviavillani@me.com (S.V.); graziella.orru@unipi.it (G.O.); angelo.gemignani@unipi.it (A.G.); ciro.conversano@unipi.it (C.C.); 2 School of Advanced Studies, University of Camerino, 62032 Camerino, Italy; 3 Department of Clinical and Experimental Medicine, University of Pisa, 56126 Pisa, Italy; mario.miniati@med.unipi.it

**Keywords:** mindfulness, MBI, MBSR, students, university, university students, mental health, psychological well-being, online meditation, distress

## Abstract

College students are particularly vulnerable to psychological distress, including anxiety, depression, and chronic stress, often triggered by academic pressure, developmental challenges, and events such as the COVID-19 pandemic. This study examined the effectiveness and feasibility of a structured mindfulness-based program—Mindfulness Laboratory (MLAB)—delivered over three academic years to psychology students in Italy through online, hybrid, and in-person formats. A total of 194 students participated, with 176 completing pre- and post-intervention assessments. Standardized self-report measures evaluated mindfulness (FFMQ, MAAS), perceived stress (PSS), resilience (RS-14), sleep quality (PSQI), depressive symptoms (BDI-II), anxiety (STAI-Y1, STAI-Y2), and self-compassion (SCS). A non-randomized control group of 51 students who did not undergo the intervention was also included. The results showed significant improvements in mindfulness, perceived stress, anxiety, and depression, with a smaller but significant increase in resilience. Sleep quality remained stable, while self-compassion levels slightly declined. Surprisingly, no significant differences were found across the three delivery formats, suggesting comparable effectiveness regardless of modality. These results support the feasibility and benefits of mindfulness-based interventions for university students. Further controlled studies with long-term follow-up are needed to confirm upon these findings.

## 1. Introduction

Over the previous 20 years, there has been a significant increase in higher education enrollment. From about 100 million students in 2000 to over 235 million in 2020, the number of students enrolled in tertiary education increased by more than 135% worldwide [1]. Between 2000 and 2023, the percentage of adults in Italy between the ages of 25 and 34 who have a tertiary degree increased by 200% [2]. Higher education can be a particularly stressful life stage, with young adults navigating academic stress and peer relationships, virtual socialization, internet addiction, financial stress and uncertainties around future careers, having to choose a field of study that will lead to a sustainable, good paying, career [3,4,5]. Consequently, symptoms of psychological distress such as anxiety, depression and perceived stress are increasingly common amongst this kind.

As a result, symptoms of mental distress such as anxiety, depression, and perceived stress are prevalent in this population. These mental health issues impact students’ well-being, academic performance, dropout rates, and quality of life [6,7]. In Italy, psychological distress is especially common among university students; a recent cross-sectional study identified 35.3% of Italian students with symptoms of anxiety, and as many as 72.9% with depressive symptoms, mostly with mild severity [8]. This issue is of particular concern among medical and health-related students. A longitudinal study conducted in Italy during the COVID-19 pandemic found that university students experienced moderate to severe symptoms of depression, anxiety, and obsessive-compulsive tendencies, with significant increases during lockdown phases, particularly among female students [9]. These patterns align with more general research on Generation Z, or those born between the middle of the 1990s and the beginning of the 2010s, which shows that they experience more psychological distress than earlier generations. Research shows that Gen Z is especially susceptible to loneliness, depression, and anxiety, partly due to economic uncertainty, anxiety related to climate change, and excessive digital exposure [10].

In the face of this mental health crisis, universities have been testing a number of interventions that seek to help prevent and reduce students’ psychological distress and build their resilience. These include but are not limited to mindfulness-based interventions (MBIs), which have garnered increasing attention as being accessible, low-cost, and having the support of the evidence base. Drawing on ancient contemplative traditions and underscored by programmatic approaches like mindfulness-based stress reduction (MBSR) and mindfulness-based cognitive therapy (MBCT), these interventions promote non-judgmental awareness of the present moment, emotional regulation, cognitive flexibility, and self-compassion [11,12]. The mechanisms promoted by mindfulness practice are particularly pertinent within the university environment, where students frequently encounter prolonged cognitive demands, performance pressures, and emotional fluctuations that test their abilities for adaptive self-regulation. In this context, various studies indicate the impact of MBIs on university students’ psychological outcomes, through the training and enhancement of mindfulness abilities (both state and trait-like). In fact, a systematic onset of reviews and meta-analysis have shown that mindfulness training reduces anxiety, depression, and stress with small to medium effect sizes [13,14]. In addition to that, it is already known that mindfulness practice enhances self-compassion, emotional regulation, and psychological well-being [15,16]. Evidence exists that the programs are effective in improving mental health in higher education settings, despite differences regarding design and delivery [17]. A recent study demonstrated that the classic 8-week mindfulness-based course was both acceptable and effective among university students, leading to improvements in mental health, well-being, and academic goal orientation, with mindfulness, resilience, and self-compassion mediating these effects [18]. Dispositional mindfulness, which refers to an individual’s natural or habitual tendency to be aware of and attentive to the present moment in a nonjudgmental way, may also be important in this context: a recent systematic review highlighted the relevance of dispositional mindfulness in youth populations, reporting consistent associations with neural activity patterns involved in emotion regulation and self-referential processing [19].

While these findings are promising, several limitations exist in current literature. Previous studies investigating mindfulness-based interventions in college students have small sample sizes, little follow-up and significant heterogeneity regarding methodology and outcome measures [13]. Moreover, only a few investigations have explored the feasibility and scalability of MBIs when delivered over extended periods or across diverse student populations. As highlighted by Dawson et al. (2020), many randomized controlled trials in this field present a high risk of bias, revealing the need for further research aimed at evaluating long-term outcomes and standardizing intervention protocols [11]. In line with this, Chiodelli et al. (2022) emphasize the frequent absence of physiological indicators and follow-up assessments, despite the encouraging results of mindfulness-based interventions (MBIs) in academic contexts [17]. To better understand the persistence of psychological benefits over time, future studies should adopt long-term follow-up designs—such as assessments at 3, 6, and 12 months—following the example of previous research in the field [20,21,22].

### Aims and Hypothesis

The main aim of this study is to evaluate how effective a structured Mindfulness Laboratory (MLAB) may be, based on the concepts of Mindfulness-Based Stress Reduction (MBSR) and specifically designed for a university environment. Our research focuses on assessing variations in important psychological factors such as mindfulness, perceived stress, resilience, anxiety, depression, sleep quality, and self-compassion, comparing the period prior to and following involvement in the intervention. The data were collected across three academic years (2021–2023), encompassing different delivery formats: online, hybrid, and in-person. Based on previous research and on the mindfulness-based interventions’ structure, our hypothesis is that participation in the MLAB leads to improvements in mindfulness (as measured by the FFMQ and MAAS), resilience (RS-14), self-compassion (SCS), and sleep quality (PSQI), as well as reductions in perceived stress (PSS), anxiety (STAI-Y), and depressive symptoms (BDI-II). Additionally, we anticipate that greater increases in mindfulness are associated with higher resilience, better sleep quality, and enhanced self-compassion. These associations are expected to reflect the role of mindfulness as a protective psychological factor.

## 2. Materials and Methods

### 2.1. Participants and Procedure

The mindfulness laboratory (MLAB) was offered as a mandatory course for second-year undergraduate psychology students at the University of Pisa. Participation was required to receive academic credit for the course. Across three academic years (2021–2022, 2022–2023, 2023–2024), a total of 194 students took part in the intervention. The delivery format varied by academic year: it was held entirely online in 2021–2022, in a hybrid format in 2022–2023 (31 students in person, 39 online), and entirely in person in 2023–2024.

The MLAB consisted of 32 h of training delivered over two weeks in December or January, with daily sessions ranging from 2 to 6 h. The structure and content of the intervention remained consistent throughout the years. The course was delivered to second-year undergraduate students during the first semester and was a mandatory component of their bachelor’s degree in psychology. At the beginning and end of the course, the participants completed a battery of psychological questionnaires via an online form.

The control group consisted of 51 first-year master’s students enrolled in the Clinical Psychology program at the University of Pisa, assessed in April–May 2022 during a regular teaching period. These students did not take part in the MLAB intervention but completed the same battery of psychological questionnaires as the intervention group. Although the groups differed in academic cycle and year of study, both were assessed outside the exam period and within a shared institutional and educational framework. All collected data included age, gender, relationship status, work status, prior experience with meditation, and frequency of mindfulness practice.

Participation in the evaluation was voluntary and anonymous. Informed consent was obtained from all participants, and the study protocol was approved by the Committee on Bioethics of the University of Pisa (Latest Review No. 2/2023 Prot. N. 0011991/2023).

### 2.2. Intervention Structure

The MLAB introduced students to core mindfulness principles and practices. The course was consistently conducted by the same instructor across all three academic years, a licensed psychotherapist and adjunct professor with extensive experience in delivering mindfulness-based interventions (author S.V.).

The theoretical portion (approximately 40%) included foundational concepts such as the definition of mindfulness, sensory awareness, reactivity cycles, acute versus chronic stress, mindful eating, automatic pilot, beginner’s mind, and mind-wandering. Emphasis was placed on group inquiry and interactive discussion.

The experiential segment (approximately 60%) comprised guided practices including lying-down and standing yoga, breath meditation, seated meditation, body scan, walking meditation, and loving–kindness (metta) meditation.

In addition to attending the in-class sessions, the participants were also assigned traditional home practice exercises adapted from the eight core classes of the Mindfulness-Based Stress Reduction (MBSR) program. The students were asked to engage in these practices throughout the course period and to submit brief written reports. The purpose of collecting these reports was solely to monitor adherence to home practice, without evaluation.

### 2.3. Measures

The participants completed the following validated self-report questionnaires at both time points (pre- and post-intervention):

- Five Facet Mindfulness Questionnaire (FFMQ) [23,24]: Assesses five key components of mindfulness—observing, describing, acting with awareness, non-judging, and non-reactivity—providing a multidimensional evaluation of dispositional mindfulness. It is one of the most widely used instruments to capture the complex structure of mindfulness and its subdimensions, with both total and facet-level analyses.
- Mindful Attention Awareness Scale (MAAS) [25,26]: Measures the general tendency to be attentive to and aware of present-moment experiences in daily life, reflecting core aspects of mindfulness such as sustained attention and absence of distraction. The 15-item unidimensional scale focuses particularly on mindlessness or lapses in attention.
- Perceived Stress Scale (PSS10) [27,28]: Evaluates the degree to which individuals appraise their lives as stressful, unpredictable, uncontrollable, and overloaded over the past month. This 10-item tool collects subjective stress responses rather than objective stressors and is sensitive to change over time.
- Resilience Scale 14 (RS-14) [29,30]: A short-form measure of resilience focusing on the measure of individuals’ ability to positively adapt to adversity, maintain purpose, and persevere through challenges. The scale is characterized by the measuring of traits such as self-reliance, meaningfulness, and equanimity.
- State-Trait Anxiety Inventory—Form Y (STAI-Y) [31,32]: Distinguishes between temporary, situational anxiety (State; Y1) and a more general, stable predisposition to anxiety (Trait; Y2). This widely used 40-item instrument separates momentary emotional responses from enduring anxious tendencies, with robust psychometric support across clinical and non-clinical populations.
- Beck Depression Inventory—Second Edition (BDI-II) [33,34]: A 21-item self-report instrument designed to assess the severity of depressive symptoms consistent with DSM-IV criteria for major depression. It includes cognitive, emotional, and somatic symptoms.
- Pittsburgh Sleep Quality Index (PSQI) [35,36]: Assesses subjective sleep quality and disturbances over the past month across seven components, including sleep latency, duration, disturbances, and daytime dysfunction. The global score differentiates between “good” and “poor” sleepers and is widely used in both clinical and community settings.
- Self-Compassion Scale (SCS) [37,38]: Measures the tendency to respond with self-kindness, a sense of common humanity, and mindfulness when facing personal difficulties or failures. The scale provides insight into how individuals treat themselves in moments of distress.

### 2.4. Analysis

All statistical analyses were performed using JASP 0.19.3.0 [39]. Descriptive statistics were calculated for all outcome variables at pre- and post-intervention. Paired-sample *t*-tests were used to assess within-group changes for the MLAB group across key psychological variables. To investigate between-group effects, a control group of students (N = 51) from the 2022 to the 2023 academic year who had not received mindfulness training was included. Group comparability at baseline was tested using independent samples *t*-tests. Although the groups differed in academic level and course year, they were assessed during comparable teaching periods (i.e., outside the exam session) within the same institutional setting. Group equivalence was statistically evaluated through independent samples *t*-tests comparing baseline characteristics across psychological and demographic variables. No significant differences were found in age, gender distribution, or in any of the main outcome variables at baseline. For each outcome variable, a 2 (Time: pre, post) × 2 (Group: MLAB vs. control) mixed-design ANOVA was conducted to assess whether the magnitude of change differed between groups. Significant Time × Group interaction effects were interpreted as evidence of differential intervention effects. Assumptions of normality and sphericity were tested and met. Moreover, multiple linear regressions were conducted to explore potential predictors of change within the MLAB group, with pre-intervention scores and demographic variables (e.g., gender, age) entered as independent variables and post-intervention scores as outcomes. Finally, we considered a reduction of ≥5 points on the BDI-II as a minimally clinically important difference (MCID), in line with distribution-based estimates; anchor-based studies further suggest a 3–6 point MID range, depending on baseline severity [40,41]. The graphs and figures were generated with the support of ChatGPT, specifically GPT-4o (“Omni”) (OpenAI, San Francisco, CA, USA). Based on statistical results processed with JASP (version 0.19.3.0), the authors requested support from ChatGPT to generate LaTeX code for data visualizations. These figures were manually reviewed and, when necessary, adjusted by the authors.

#### Power Analysis

An a priori power analysis was conducted using G*Power (version 3.1) [42] for a one-way ANOVA with three groups, assuming a medium effect size (f = 0.25), α = 0.05, and power = 0.80. The analysis indicated a required total sample of 158 participants (Figure S1).

## 3. Results

### 3.1. Descriptive Statistics; Sociodemographic and Initial Information

Descriptive statistics were computed for the main socio-demographic and mindfulness-related variables. Out of 194 identified participants, 176 (90.7%) completed both pre- and post-intervention assessments and were included in the final analysis. These eighteen missing participants (9.3%) were excluded due to incomplete data or unmatchable identifiers across timepoints.

Socio-demographic information was available for 175 participants of MLAB and for 51 controls. The breakdown of baseline demographic and psychological measures for both MLAB and control groups is reported in Supplementary Tables S1–S3. In the MLAB groups, the majority were female (85.1%), with a mean age of 22.34 years (SD = 4.94, range = 19–56). Most participants were single (M = 1.07, SD = 0.33; 1 = single), and not currently engaged in paid work (M = 1.69, SD = 1.16; 1 = full-time student). Regarding meditation experience, 24% reported having practiced meditation at least once in their lives although regular practice was uncommon. The frequency of practice was generally low, with most participants reporting they had never practiced or only a few times per year. In the control group, the participants displayed a similar socio-demographic profile: the majority were also single and full-time students with low meditation experience (74.5%).

**Figure 1:**
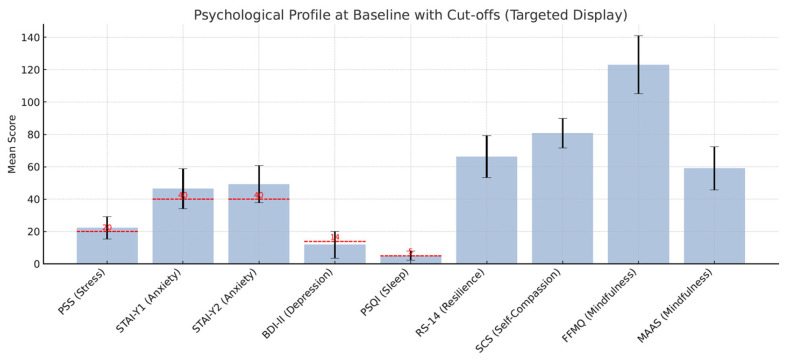
Psychological profile at baseline with clinical cut-offs. Baseline mean scores for perceived stress (PSS), anxiety (STAI-Y1, STAI-Y2), depression (BDI-II), sleep quality (PSQI), resilience (RS-14), self-compassion (SCS), and mindfulness (FFMQ, MAAS) in this sample. Clinical cut-off thresholds were indicated where possible.

At baseline, the sample displayed moderate levels of psychological distress [Figure 1]. The average score on the Perceived Stress Scale (PSS) was 22.22 (SD = 6.86), which falls within the moderate stress range. Anxiety levels were also elevated, with a mean score of 46.50 (SD = 12.30) on the STAI-Y1 and 49.29 (SD = 11.52) on the STAI-Y2. Symptoms of depression, as measured by the BDI-II, showed a mean score of 11.81 (SD = 8.36), indicating mild to moderate depression. Sleep quality (PSQI) was impaired, with a mean score of 5.12 (SD = 2.75), above the clinical cut-off of 5. Resilience scores (RS-14) averaged 66.28 (SD = 12.89), and self-compassion (SCS) was moderate with a mean of 80.72 (SD = 9.10). Baseline mindfulness levels were relatively low, with a mean of 123.01 (SD = 17.85) on the FFMQ and 58.96 (SD = 13.35) on the MAAS.

### 3.2. Pre–Post Comparison

The Shapiro–Wilk test was used to evaluate the normality of the distributions. While certain variables exhibited differences from normality, paired samples *t*-tests were performed since they are regarded as resilient to minor breaches of this assumption, especially in moderately sized samples (N = 175).

**Figure 2:**
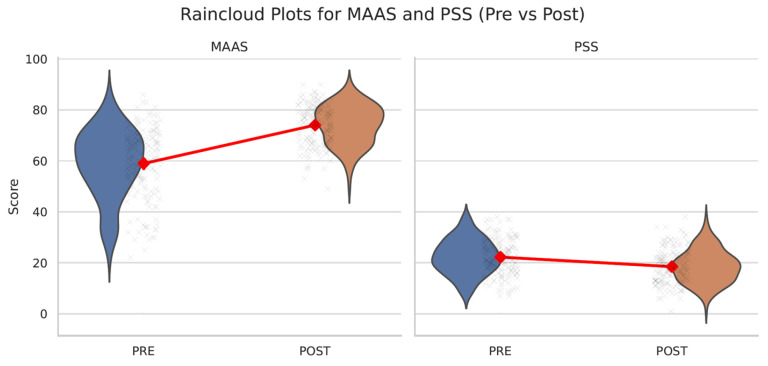
Raincloud plots for MAAS and PSS (pre- vs. post-intervention). Raincloud plots showing pre- and post-intervention scores on the Mindful Attention Awareness Scale (MAAS) and the Perceived Stress Scale (PSS). Significant improvements were observed in both outcomes.

The results indicated significant improvements across all subscales of the Five Facet Mindfulness Questionnaire (FFMQ), with the strongest effect observed for the Observing facet (t (174) = −9.83, *p* < 0.001, d = −0.682). Total FFMQ scores increased significantly (t (174) = −10.21, *p* < 0.001, d = −0.670), as did MAAS scores (t (174) = −15.26, *p* < 0.001, d = −1.340), indicating enhancement in mindfulness. Perceived stress decreased significantly (t (174) = 7.31, *p* < 0.001, d = 0.554), as did anxiety levels on both STAI-Y1 and STAI-Y2 (*p* < 0.001). Depression symptoms, measured by the BDI-II, also showed a significant reduction (t (174) = 8.06, *p* < 0.001, d = 0.446). A modest yet significant increase was observed in resilience (RS-14: t (174) = −4.04, *p* < 0.001, d = −0.221). Sleep quality (PSQI) did not show a significant change (t (174) = 0.01, *p* = 0.994), while self-compassion scores (SCS) decreased slightly but significantly (t (174) = 4.35, *p* < 0.001, d = 0.307). To further illustrate the effects of the intervention, representative raincloud plots are reported in Figure 2: for brevity, only two of the most significant outcomes are shown (MAAS and PSS).

### 3.3. Correlations Analysis

To investigate the connections between psychological changes, Pearson’s correlations were calculated based on the differences (Δ) before and after for all main outcome measures. The results indicate that improvements in mindfulness, measured by both FFMQ and MAAS, have a strong link to decreases in perceived stress, as well as reductions in anxiety (both state and trait) and symptoms of depression. For instance, changes in FFMQ were negatively correlated with changes in trait anxiety (r = −48) and perceived stress (r = −41). Improvements in resilience (RS-14) were also associated with reductions in depressive symptoms (r = −40). The complete matrix of Pearson’s correlations among psychological change scores is available in Supplementary Table S4.

### 3.4. Comparison of Pre–Post Changes by Intervention Delivery Mode

A one-way ANOVA was conducted to examine whether the effectiveness of the intervention differed by course format (online, hybrid, in-person). The analysis revealed no statistically significant differences among the three groups across any of the outcome measures. This includes mindfulness (FFMQ: F(2, 172) = 1.08, *p* = 0.343; MAAS: F = 0.89, *p* = 0.413), stress (PSS: F = 0.24, *p* = 0.788), resilience (RS-14: F = 0.88, *p* = 0.416), anxiety (STAI-Y1: F = 0.73, *p* = 0.485; STAI-Y2: F = 0.91, *p* = 0.404), depression (BDI-II: F = 0.67, *p* = 0.514), sleep quality (PSQI: F = 0.63, *p* = 0.531), and self-compassion (SCS: F = 0.43, *p* = 0.651).

#### Pre–Post Changes Within Each Cohort

To further explore potential differences, we examined pre–post changes within each delivery format separately. All three cohorts—online, blended, and in-person-showed consistent improvements in mindfulness, perceived stress, and awareness. The FFMQ scores increased from M = 125.4 (SD = 18.0) to M = 135.2 (SD = 20.1) in the online group, from M = 122.9 (SD = 17.6) to M = 134.8 (SD = 16.0) in the blended group, and from M = 120.2 (SD = 17.9) to M = 134.1 (SD = 14.6) in the in-person group. The PSS scores decreased from M = 22.4 (SD = 7.3) to M = 19.2 (SD = 6.7), M = 22.5 (SD = 7.2) to M = 18.0 (SD = 6.0), and M = 21.8 (SD = 6.1) to M = 18.1 (SD = 7.0), respectively. The MAAS scores increased from M = 59.9 (SD = 13.4) to M = 73.1 (SD = 7.9) in the online group, from M = 60.1 (SD = 13.2) to M = 74.9 (SD = 7.5) in the blended group, and from M = 56.8 (SD = 13.5) to M = 74.3 (SD = 8.0) in the in-person group. Parallel trends were observed across other outcome variables. RS-14 scores increased similarly in all groups (online: M = 66.6 to 67.9; blended: 66.4 to 69.9; in-person: 65.8 to 69.8). Anxiety (STAI-Y1) decreased in all cohorts (from M = 47.1 to 43.3, 45.7 to 41.1, and 46.5 to 41.0, respectively), as did trait anxiety (STAI-Y2), with reductions from M = 49.4 to 47.4 in the online group, 48.8 to 46.3 in the blended group, and 49.6 to 45.5 in the in-person group. The BDI-II scores also decreased from M = 11.6 to 8.6 (online), 11.6 to 7.7 (blended), and 12.0 to 8.4 (in-person). Although the PSQI scores showed slight fluctuations, overall patterns suggested improved sleep quality. Finally, self-compassion scores (SCS) remained stable or slightly decreased, consistent with prior findings on early-stage mindfulness interventions.

### 3.5. Between-Group Comparisons

**Figure 3:**
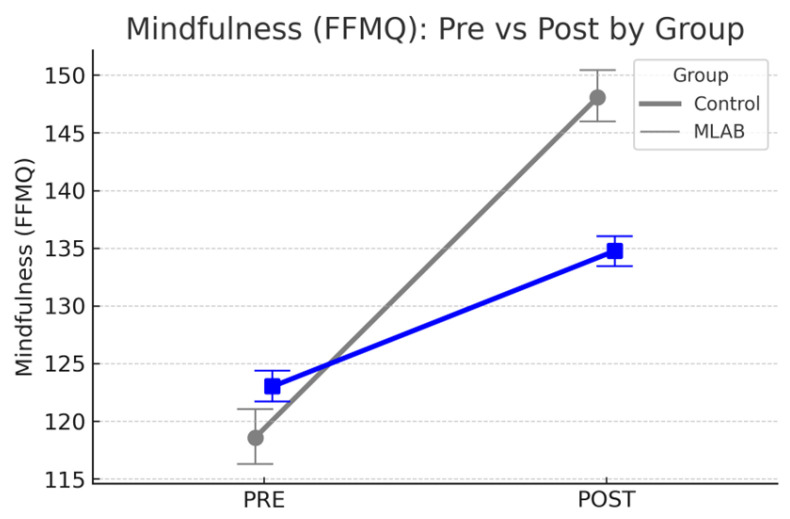
Changes in psychological outcomes from pre- to post-intervention for the MLAB and control groups. The MLAB group showed significantly greater improvements in mindfulness (FFMQ), perceived stress (PSS), state anxiety (STAI-Y1), and depressive symptoms (BDI-II) compared to the control group. Error bars represent 68% confidence intervals. The blue line represents the control group.

To evaluate the impact of the intervention beyond time-related effects, mixed-design ANOVAs were performed comparing the MLAB group to a non-randomized control group. All outcome variables were analyzed using a 2 (Time: pre, post) × 2 (Group: MLAB vs. control) design. Significant Time × Group interaction effects were observed for mindfulness (FFMQ: *p* < 0.001; MAAS: *p* < 0.001), perceived stress (PSS: *p* < 0.001), state anxiety (STAI-Y1: *p* < 0.001), trait anxiety (STAI-Y2: *p* = 0.012), and depressive symptoms (BDI-II: *p* < 0.001), indicating significantly greater improvements in the intervention group compared to controls. No significant interaction effects emerged for resilience (RS-14: *p* = 0.35), self-compassion (SCS: *p* = 0.59), or sleep quality (PSQI: *p* = 0.38), suggesting that changes in these outcomes were either similar between groups or negligible overall. Full statistical results are provided in Supplementary Table S5. Figure 2 and Figure 3 illustrate representative comparisons of the most significant outcomes.

### 3.6. Predictors of Stress Reduction Following the Intervention

**Figure 4:**
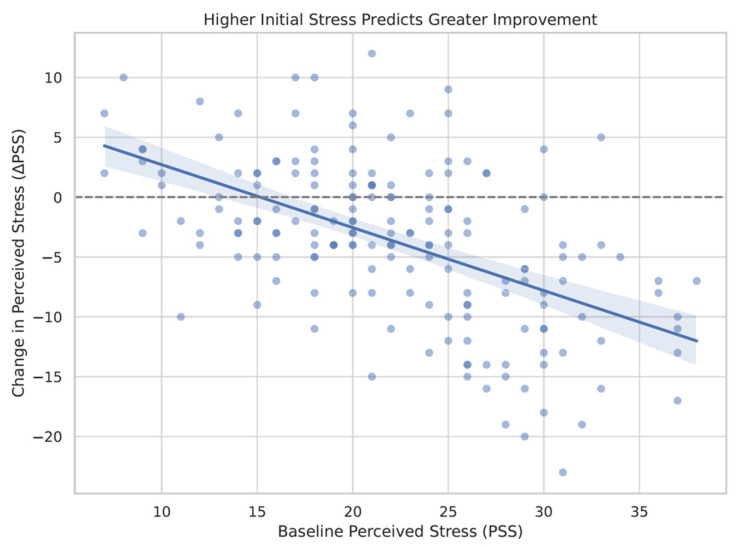
Baseline stress predicts greater improvement. Scatter plot depicting the relationship between baseline perceived stress scores (PSS) and changes in stress following the intervention (ΔPSS). Higher initial stress was associated with greater reductions in perceived stress. The darker blue dots indicate higher baseline perceived stress scores, whereas the lighter blue dots reflect lower initial stress levels.

A multiple linear regression was conducted to identify predictors of change in perceived stress (ΔPSS) following the intervention. The model included baseline stress (PSS), mindfulness (MAAS, FFMQ), meditation experience, age, gender, and course format as predictors. The overall model was significant (R^2^ = 0.34, F(7, 167) = 12.53, *p* < 0.001). Results indicated that baseline stress levels were a strong negative predictor of change (β = −0.65, *p* < 0.001), suggesting that individuals with higher initial stress tended to show greater reductions. Additionally, gender was a significant predictor (β = 2.50, *p* = 0.041), with female participants reporting greater stress reduction than males. This relationship is visually depicted in Figure 4, which shows that higher baseline stress scores were associated with greater decreases in perceived stress following the intervention.

### 3.7. Proportion of Participants Exhibiting Clinically Significant Change

With the aim of evaluating clinically meaningful improvements, standard cut-offs were used to identify participants with elevated baseline scores in depression (BDI-II), sleep disturbances (PSQI), perceived stress (PSS), and anxiety (STAI-Y1, STAI-Y2). The Δ ≥ 5 criterion aligns with clinical thresholds defined in prior research as representing meaningful change. Among the participants initially above the clinical threshold for depression (n = 66; 37.7%), 33 (50%) moved below the threshold following the intervention, and 64 (97%) showed a reduction of at least five points. Similar patterns emerged for perceived stress, with 116 participants initially above the threshold, 50 of whom moved below the threshold and 69 exhibited a reduction of five or more points. Improvements were also observed for anxiety measures. In contrast, although all participants exceeded the clinical cut-off for poor sleep quality at baseline (PSQI > 5), only 59 reported a reduction of at least five points, and none dropped below the clinical threshold.

### 3.8. Qualitative Feedback

To complement the quantitative outcomes, we collected open-ended responses from participants regarding their subjective experience of the course. Most students reported that their expectations were either met or exceeded, especially in terms of gaining greater awareness of the body–mind connection and learning practical strategies to manage stress. The most appreciated practices included the body scan, sitting meditation, and yoga, which were often described as effective in fostering relaxation, concentration, and bodily awareness. Conversely, some participants found the body scan or seated practices more challenging, due to difficulty maintaining attention or physical discomfort. Despite these individual differences, the vast majority expressed an intention to continue practicing mindfulness, either regularly or occasionally. Many emphasized a sense of personal benefit and expressed hope that similar courses would be offered again in the future. The full set of translated (Italian to English) open-ended qualitative responses is included in the Supplementary Materials.

## 4. Discussion

### 4.1. About the Effectiveness of the Intervention

The present study evaluated the effectiveness of a structured mindfulness intervention—Mindfulness Laboratory (MLAB)—in reducing psychological distress among undergraduate psychology students in Italy, knowing that previous mindfulness interventions targeting university students have shown comparable effects across various academic disciplines and cultural contexts [20,43].

At baseline, the participants displayed moderate to elevated levels of distress, including high perceived stress and anxiety, mild to moderate depressive symptoms, poor sleep quality, and relatively low levels of dispositional mindfulness. Sadly, the mean scores for both state and trait anxiety exceeded normative values for non-clinical young adults, and perceived stress levels reflected a pervasive sense of pressure and overload. This psychological profile supports the prior literature identifying university students as a population particularly vulnerable to emotional dysregulation and burnout [8,9], as confirmed by studies reporting similar symptoms in non-clinical student populations prior to intervention, highlighting the importance of early, scalable, and preventive interventions [6,7].

Following participation in the MLAB course, the students exhibited significant improvements across multiple psychological domains. Dispositional mindfulness, measured through both the FFMQ and MAAS, increased substantially. The most notable gains occurred in the “observing” and “acting with awareness” dimensions of the FFMQ, which are closely related to attentional control and present-moment awareness—two foundational mechanisms in mindfulness-based interventions. These improvements are especially relevant for university students, who frequently contend with cognitive overload and emotional distraction [11]. These findings are in line with previous studies showing that even brief or app-delivered mindfulness interventions can foster attention regulation and present-moment awareness in student populations [44]. They are also consistent with results from de Bruin and colleagues (2015), who observed significant improvements in awareness and attentional control following a low-intensity mindfulness program delivered within a university curriculum [45]. Our students’ feedback reinforced this evidence: several reported having learned to integrate short pauses for mindful breathing during study sessions, which they described as enhancing focus and reducing stress. Such micro-practices reflect the successful translation of mindfulness principles into daily academic behavior and may contribute to long-term cognitive resilience.

Significant reductions were also found in perceived stress and in both state and trait anxiety. These findings are consistent with previous studies demonstrating that practicing mindfulness can help interrupt ruminative thinking and promote more adaptive cognitive reappraisal strategies [15,17]. Moreover, qualitative responses are informative about the extent to which anxiety affects students’ daily lives. This aligns with a growing body of literature showing that anxiety disorders represent one of the most common psychological challenges among young adults and are linked to both academic impairment and decreased quality of life [14].

In line with existing evidence on the mood-regulating effects of MBIs [13], the intervention also led to a meaningful reduction in depressive symptoms. This effect may be explained by the cultivation of non-judgmental awareness and emotional acceptance promoted by mindfulness, which helps reduce negative self-referential thinking and facilitates a more balanced response to internal experiences. Other studies showed that brief mindfulness sessions in university settings can significantly reduce depression levels [46,47,48].

Participating in the course also produced a modest but significant increase in resilience. This psychological resource is increasingly recognized as a dynamic capacity influenced by both personal and environmental factors. Mindfulness practices may enhance resilience by reducing impulsive reactivity, encouraging acceptance of difficult situations, and improving present-focused awareness [49]. Even small improvements in this domain may yield substantial long-term benefits for students facing recurring stressors.

Conversely, a small but significant decline in self-compassion was observed post-intervention. While unexpected, this finding may reflect a transient increase in self-critical awareness during the early phases of mindfulness practice. This has been reported in prior literature, where initial exposure to contemplative self-observation sometimes leads to greater contact with unresolved self-judgment [37,50]. It is also worth noting that although the MLAB included some loving–kindness elements, it was not explicitly designed to train self-compassion. Targeted programs such as Mindful Self-Compassion (MSC) may be better suited to cultivating this specific dimension.

Lastly, no significant change was observed in sleep quality. This result is in line with other studies suggesting that sleep difficulties may require more specific or longer-term interventions [51,52]. Nevertheless, previous work by our team has shown that mindfulness-based interventions may positively affect sleep quality in clinical populations, indicating that the impact of mindfulness on sleep may vary depending on baseline severity and context [53].

### 4.2. Delivery Format

A key strength of the present study lies in its naturalistic implementation of the mindfulness intervention across three consecutive cohorts, each receiving the MLAB course in a different delivery format: online, hybrid, or fully in-person. Despite differences in context and modality, no significant variations in intervention effectiveness were observed between groups on any of the outcome measures.

This absence of group differences was not fully expected. Previous research has suggested that the mode of delivery can influence both engagement and outcomes in mindfulness-based programs. For example, in-person interventions have often been associated with higher participant satisfaction, group cohesion, and greater depth of experiential learning, while online formats are at risk for a lower adherence and emotional engagement [20,54]. Meta-analytic data have indicated that web-based MBIs may show smaller effect sizes compared to in-person ones, especially for stress and depressive symptoms [20]. Conversely, a smaller body of research suggests that digitally delivered mindfulness interventions—including those utilizing virtual assistants, chatbots, or mobile applications—can be as effective as human-facilitated telehealth formats [55,56,57].

The uniformity of outcomes across formats in our study may be attributed to the structured and immersive nature of the MLAB program. Unlike many optional or self-guided interventions, participation in MLAB was mandatory for course credit, and included substantial experiential content—both in live sessions and through assigned home practice. The students were required to submit short written reflections on their daily practice, which may have reinforced accountability and maintained engagement regardless of the delivery format. Additionally, all sessions, whether remote or in-person, were led by the same experienced instructor, ensuring consistency in delivery, tone, and style.

When properly designed and integrated into the academic framework, mindfulness interventions can be delivered effectively across different formats without compromising outcomes. This has important implications for the sustainability of mental health promotion programs in university settings; in particular, remote and hybrid modalities may allow institutions to reach broader populations, reduce logistical barriers, and adapt flexibly to emerging needs, including post-pandemic educational demands. Future research could investigate which specific elements—such as group interaction, instructor presence, or self-monitoring tasks—most strongly contribute to intervention efficacy in different formats.

### 4.3. Predictors of Change and Clinically Meaningful Improvement

A multiple linear regression analysis was conducted to identify predictors of stress reduction following the intervention. The model revealed that higher baseline levels of perceived stress were the strongest predictor of post-intervention improvement, with individuals reporting greater initial distress showing the most pronounced reductions in stress. This finding is consistent with a well-documented “room for improvement” effect, whereby participants with more severe baseline symptoms often benefit most from psychological interventions [21]. This may suggest that mindfulness training could be particularly valuable as an early intervention for those students who experience the highest psychological burden.

Gender also emerged as a significant predictor, with female participants reporting greater reductions in perceived stress compared to their male counterparts. While the underlying mechanisms remain unclear, this result are in line previous findings suggesting that women may engage more deeply in emotion-focused coping strategies such as mindfulness and may derive greater benefit from interventions that emphasize emotional awareness and regulation [58,59]. These differences may also reflect gendered patterns in emotional disclosure, motivation, and help-seeking behaviors. In the Italian academic context, prior studies have shown that female university students tend to experience a higher emotional and relational load, often balancing academic demands with social and caregiving expectations [60,61]. These sociocultural factors may not only heighten initial distress but also promote greater receptivity to interventions like mindfulness, which encourage internal attunement and self-regulation. Lastly, while gender was a significant predictor in the regression analysis, this result must be interpreted with caution due to the strong predominance of female participants in the sample.

Our data also indicate substantial improvement in participants who began the intervention above clinical thresholds; among students with elevated levels of depressive symptoms, 50% no longer met the cut-off for mild depression at post-intervention, and nearly all showed a decrease of five points or more on the BDI-II. Similar patterns were observed for perceived stress, with 60% of students above the clinical cut-off showing at least a five-point reduction, and 43% falling below the threshold after the intervention. Reductions were also noted in both state and trait anxiety, reinforcing the intervention’s effectiveness in targeting multiple facets of distress.

Conversely, although all participants scored above the clinical threshold for poor sleep quality at baseline, no one fell below the cut-off at post-intervention. While a modest proportion of students did report improvements in sleep (defined as a reduction of at least five points on the PSQI), the lack of substantial clinical shift highlights the complexity of addressing sleep disturbances through general mindfulness training alone. As already stated, targeted sleep-focused interventions may be necessary to produce clinically significant changes in this domain.

### 4.4. Limitations and Future Directions

While the present study offers encouraging evidence regarding the effectiveness of mindfulness training in university students, several limitations must be acknowledged.

The first limitation could be that, although a control group was included for comparison, the lack of random assignment and the fact that the control group was drawn from a different academic year and course limit the ability to make strong causal inferences. While the baseline characteristics were largely comparable, potential historical, contextual, or curricular differences between cohorts may have influenced outcomes. Second, all measures relied on self-report questionnaires, which are susceptible to biases; this limitation is especially relevant in mandatory university courses, where students may feel compelled to report perceived benefit. While anonymity was maintained to reduce this risk, future research would benefit from the inclusion of behavioral indicators (e.g., academic performance, physiological stress markers, or digital tracking of practice adherence). Third, the sample was composed almost entirely of female psychology students, limiting the generalizability of the findings. Gender imbalance, while common in Italian psychology programs, may obscure meaningful differences in intervention responsiveness and emotional processing across genders.

A further limitation concerns the lack of follow-up assessment. While short-term benefits were clearly demonstrated, the durability of these effects remains unknown. Longitudinal follow-up is essential to determine whether gains in mindfulness and psychological well-being persist over time, particularly under the pressure of exams, transitions, or post-graduate stressors.

## 5. Conclusions

The present research shows that a short, organized mindfulness program integrated into the university courses can significantly decrease psychological distress while improving dispositional mindfulness and resilience among psychology undergraduate students. This effect was particularly pronounced in students with higher initial stress levels. Consistent improvements were seen in all delivery formats, indicating that the program is likely to be adapted for online delivery.

## Data Availability

The data presented in this study are available on request from the corresponding author. The data are not publicly available due to privacy and ethical restrictions.

## Supplementary Materials

The following supporting information can be downloaded at https://www.mdpi.com/article/10.3390/ijerph22071027/s1. Supplementary File S1 includes the following: Figure S1: Power Analysis; Table S1: Sociodemographic variables by delivery format in the MLAB group; Table S2: Sociodemographic variables in the control group; Table S3: Baseline characteristics and pre-intervention scores in the MLAB and control groups; Table S4: Correlations between pre–post changes in psychological measures; Table S5: Time × Group interaction effects from mixed-design ANOVAs; and the complete set of written qualitative responses, voluntarily provided by participants and translated from Italian to English.
