# Supplementary material for: Addressing Psychological Distress in College Students Through Mindfulness Training: A Pre–Post Intervention Across Three Cohorts with Different Delivery Methods
Source: Int J Environ Res Public Health. 2025 Jun 27;22(7):1027. doi: 10.3390/ijerph22071027 (PMC12294724; doi:10.3390/ijerph22071027)
Supplement: Supplementary file 1 [file ijerph-22-01027-s001.zip › ijerph-3659856-supplementary.pdf]

## Supplementary Figure:

### Fig.S1 Power Analysis

*Power curve for a one-way ANOVA with three groups, assuming a medium effect size (f = 0.25) and α = 0.05. The red dashed line marks the conventional 80% power threshold, and the green dashed line indicates the sample size of the present study (N = 175), which exceeds the required minimum (N = 158) to detect medium effects with adequate statistical power.*

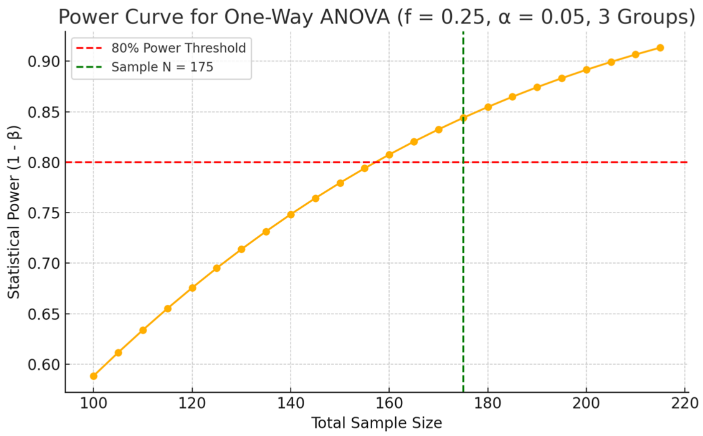

**Supplementary Tables**

**Table S1.**

*Sociodemographic variables by delivery format in the MLAB group (n = 175). Frequencies and percentages are reported for each category across online, hybrid, and in-person formats. The final column shows total values aggregated across formats.*

| Variable | Online | Hybrid | In-person | Total |
|---|---|---|---|---|
| Married with previous marriage(s) | 0 (0.0%) | 1 (1.9%) | 0 (0.0%) | 1 (0.6%) |
| Married or cohabiting | 5 (7.6%) | 1 (1.9%) | 1 (1.8%) | 7 (4.0%) |
| Separated or divorced | 0 (0.0%) | 1 (1.9%) | 0 (0.0%) | 1 (0.6%) |
| Single | 61 (92.4%) | 51 (94.4%) | 54 (98.2%) | 166 (94.9%) |
| Full-time student | 49 (74.2%) | 37 (68.5%) | 39 (70.9%) | 125 (71.4%) |
| Student with occasional job | 11 (16.7%) | 6 (11.1%) | 10 (18.2%) | 27 (15.4%) |
| Student with full-time job | 2 (3.0%) | 3 (5.6%) | 1 (1.8%) | 6 (3.4%) |
| Student with part-time job | 4 (6.1%) | 8 (14.8%) | 5 (9.1%) | 17 (9.7%) |
| No meditation experience | 54 (81.8%) | 43 (79.6%) | 36 (65.5%) | 133 (76.0%) |
| With meditation experience | 12 (18.2%) | 11 (20.4%) | 19 (34.5%) | 42 (24.0%) |

**Table S2.**

*Sociodemographic variables in the control group (n = 51).*

| Variable | Control group (n = 51) |
|---|---|
| Married with previous marriage(s) | 0 (0.0%) |
| Married or cohabiting | 5 (9.8%) |
| Separated or divorced | 0 (0.0%) |
| Single | 46 (90.2%) |
| Full-time student | 35 (68.6%) |
| Student with occasional job | 2 (3.9%) |
| Student with full-time job | 8 (15.7%) |
| Student with part-time job | 6 (11.8%) |
| No meditation experience | 38 (74.5%) |
| With meditation experience | 13 (25.5%) |

**Table S3.**

*Baseline characteristics and pre-intervention scores in the MLAB and control groups.*

| Group | Age (mean ± SD) | Gender (M/F) | FFMQ (PRE) | MAAS (PRE) | PSS (PRE) | STAI-Y1 (PRE) | STAI-Y2 (PRE) | BDI (PRE) | RS-14 (PRE) | SCS (PRE) | PSQI (PRE) |
|---|---|---|---|---|---|---|---|---|---|---|---|
| Control (n=51) | 24.04 ± 5.64 | 13/38 | 118.59 ± 17.41 | 57.84 ± 14.18 | 21.53 ± 6.39 | 48.63 ± 10.44 | 49.96 ± 12.46 | 10.51 ± 7.2 | 68.55 ± 11.96 | 80.94 ± 7.58 | 15.49 ± 8.35 |
| MLAB (n=176) | 22.34 ± 4.94 | 26/149 | 123.01 ± 17.85 | 58.96 ± 13.35 | 22.22 ± 6.86 | 46.5 ± 12.29 | 49.29 ± 11.52 | 11.81 ± 8.36 | 66.28 ± 12.89 | 80.72 ± 9.1 | 35.12 ± 27.49 |

**Table S4.**

*Correlations Between Pre-Post Changes in Psychological Measures*

| **Variable 1** | **Variable 2** | **Pearson's r** |
|---|---|---|
| Δ Anxiety (Trait) | Δ Perceived Stress | 0.48*** |
| Δ Anxiety (State) | Δ Anxiety (Trait) | 0.45*** |
| Δ Depression | Δ Anxiety (Trait) | 0.40** |
| Δ Depression | Δ Anxiety (State) | 0.40** |
| Δ Perceived Stress | Δ Anxiety (State) | 0.38** |
| Δ Depression | Δ Perceived Stress | 0.37** |
| Δ Mindfulness (FFMQ) | Δ Mindfulness (MAAS) | 0.32** |
| Δ Resilience | Δ Mindfulness (FFMQ) | 0.29* |
| Δ Depression | Δ Mindfulness (FFMQ) | -0.25* |
| Δ Depression | Δ Mindfulness (MAAS) | -0.25* |
| Δ Anxiety (State) | Δ Resilience | -0.26* |
| Δ Anxiety (Trait) | Δ Resilience | -0.29* |
| Δ Anxiety (State) | Δ Mindfulness (FFMQ) | -0.33** |
| Δ Anxiety (State) | Δ Mindfulness (MAAS) | -0.38** |
| Δ Resilience | Δ Depression | -0.40** |
| Δ Mindfulness (FFMQ) | Δ Perceived Stress | -0.41** |
| Δ Anxiety (Trait) | Δ Mindfulness (FFMQ) | -0.48*** |

**Table S5.**

*Time × Group interaction effects from mixed-design ANOVAs.*

| Variable | Time × Group p-value |
|---|---|
| Mindfulness (trait/state) (FFMQ) | < .001 |
| Perceived Stress (PSS) | < .001 |
| State Anxiety (STAI-Y1) | < .001 |
| Depressive Symptoms (BDI-II) | < .001 |
| Mindfulness (trait) (MAAS) | < .001 |
| Trait Anxiety (STAI-Y2) | 0.0122 |
| Resilience (RS-14) | 0.3546 |
| Sleep Quality (PSQI) | 0.3817 |
| Self-Compassion (SCS) | 0.5870 |
